# Effects of Different Vegetable Oils on the Nonalcoholic Fatty Liver Disease in C57/BL Mice

**DOI:** 10.1155/2023/4197955

**Published:** 2023-01-14

**Authors:** Camila Sanches Manca, Lívia Maria Cordeiro Simões-Ambrosio, Paula Payão Ovídio, Leandra Zambelli Ramalho, Alceu Afonso Jordao

**Affiliations:** ^1^Department of Internal Medicine, Faculty of Medicine of Ribeirão Preto, University of São Paulo, Av. Bandeirantes 3900, Ribeirão Preto, SP 14049-900, Brazil; ^2^Department of Pathology, Faculty of Medicine of Ribeirão Preto, University of São Paulo, Ribeirão Preto, São Paulo, Brazil; ^3^Department of Health Sciences at Ribeirão Preto Medical School, University of Sao Paulo, Ribeirão Preto-SP 14049-900, Brazil

## Abstract

**Background:**

Nonalcoholic fatty liver disease (NAFLD) is the most common hepatic disorder, affecting 22–28% of the adult population and more than 50% of obese people all over the world. Modulation of the fatty acids in diet as a means of prevention against nonalcoholic fatty liver disease in animal models (NAFLD) remains unclear. The treatment of NAFLD has not been described in specific guidelines so far. Thus, the justification for the study is to check modifications in macronutrients composition, fatty acids, in particular, play a significant role in the treatment of NAFLD regardless of weight loss.

**Aim:**

To investigate different vegetable oils in prevention and progression of NAFLD in animal models.

**Methods:**

For the experiment were used fifty C57BL/6J mice male fed with high fat and fructose diet (HFD) to induce the NAFLD status and they received different commercial vegetable oils for 16 weeks to prevent steatosis. Liver steatosis and oxidative stress parameters were analyzed using biochemical and histological methods. Fatty acids profile in the oils and in the liver samples was obtained.

**Results:**

The high fat and fructose diet led to obesity and the vegetable oils offered were effective in maintaining body weight similar to the control group. At the end of the experiment (16 weeks), the HFHFr group had a greater body weight compared to control and treated groups (HFHFr: 44.20 ± 2.34 g/animal vs. control: 34.80 ± 3.45 g/animal; *p* < 0.001; HFHFr/OL: 35.40 ± 4.19 g/animal; HFHFr/C: 36.10 ± 3.92 g/animal; HFHFr/S: 36.25 ± 5.70 g/animal; *p* < 0.01). Furthermore, the HFD diet has caused an increase in total liver fat compared to control (*p* < 0.01). Among the treated groups, the animals receiving canola oil showed a reduction of hepatic and retroperitoneal fat (*p* < 0.05). These biochemical levels were positively correlated with the hepatic histology findings. Hepatic levels of omega-3 decreased in the olive oil and high fat diet groups compared to the control group, whereas these levels increased in the groups receiving canola and soybean oil compared to control and the high fat groups.

**Conclusion:**

In conclusion, the commercial vegetable oils either contributed to the prevention or reduction of induced nonalcoholic fatty liver with high fat and fructose diet, especially canola oil.

## 1. Introduction

Nonalcoholic fatty liver disease (NAFLD) is the most common hepatic disorder, affecting 22–28% of the adult population and more than 50% of obese people all over the world [1, 2]. NAFLD is characterized by abnormal fat accumulation in the liver (simple steatosis), which can be reversed by changes in lifestyle. The progression of NAFLD to nonalcoholic steatohepatitis (NASH) covers a broad spectrum ranging from NAFLD with development of inflammatory changes to possible cirrhosis and cellular hepatocarcinoma. NAFLD/NASH is strongly associated with metabolic abnormalities such as obesity, insulin resistance, and dyslipidemia [3–7]. The mechanisms leading to disease progression and the treatment required have not been fully clarified.

Recent studies have focused on primary physiopathology of NAFLD and on mechanisms leading to NASH, fibrosis, and hepatocyte injury that have not been fully clarified although it may be stated that the cause of these conditions is polygenic and multifactorial. There is an association between genes and the participation of the environment, alongside the diet and sedentarism, whose importance has been demonstrated [8, 9].

The currently most accepted mechanism is the classical two-hit theory. The first hit is characterized by steatosis resulting from an imbalance in the formation and turnover of triacylglycerides, and it is believed to be affected by insulin resistance. The second hit involves oxidative stress and inflammatory cytokines production leading to hepatic injury [5, 10]. The role of diet in physiopathology and in the progression of NAFLD is not clear. A high-calorie diet, excess of fatty acids (especially saturated ones), and high simple sugars intake, with greater importance on fructose and saccharose, have seemed to be implicated in the disease development. Fructose is used as a food preservative, mainly present in sugar-sweetened beverages, and it is known for stimulating *de novo* lipogenesis and increasing liver oxidation/inflammation [11–13]. In addition to fructose/saccharose, the consumption of saturated fatty acids is implicated in the pathogenesis of NAFLD [13, 14]. Despite obtaining contradictory results, specific fatty acids have been proposed to be involved in hepatic lipogenesis, possibly acting on the induction or reversal of the signs and symptoms of NAFLD [15–18].

The treatment of NAFLD has not been described in specific guidelines so far. Lifestyle changes are the primary disease treatment option and weight loss is strongly recommended for NAFLD patients. However, recent studies have demonstrated that modifications in macronutrient composition and fatty acids, in particular, play a significant role in the treatment of NAFLD regardless of weight loss [15–18]. An increase in saturated fat intake is usually associated with increased insulin resistance, which may induce the progression of NAFLD, whereas monounsaturated (MUFA) and polyunsaturated (PUFA) fatty acids may prevent it by inhibiting genes involved in the mechanisms of *de novo* lipogenesis [13]. Using different n-3, n-7, n-6, and n-9-rich lipid formulations, Siddiqui et al. [13] demonstrated NAFLD reduction.

The aim of this present study was settled in order to investigate the effect of different vegetable oils on the prevention of NAFLD.

## 2. Materials and Methods

### 2.1. Animals and Diet

Male C57/B6J mice weighing 20 g at the beginning of the experiment were obtained from the Central Animal House of the Faculty of Medicine of Ribeirão Preto (FMRP), University of São Paulo, and maintained under controlled conditions of temperature (22 ± 2°C) and of humidity and on a light (7:00 am–7:00 pm)/dark (7:00 pm–7:00 am) cycle. Water and food were supplied *ad libitum*. Animals were handled according to Brazilian College of Animal Experimentation recommendations and all procedures were approved by the Ethics Committee of FMRP (protocol no. 017/2015, March 30, 2015). Animals were randomly assigned to five experimental groups. The control group (CONT) received the AIN-93 diet for growth containing 20% protein (casein), 63% carbohydrates (53% cornstarch and 10% saccharose), 7% fat (soybean oil), 5% fiber, 3.5% AIN-93G mineral mix, 1% vitamin mix, 0.3% L-cysteine, 0.25% choline, and 0.002% di-terc-butyl methyl phenol [19]. Treated groups received a modified AIN-95 diet for growth in which lipid sources were elevated and all carbohydrate sources were replaced by fructose. The high-lipid + fructose group received western type diet containing 50% fat (lard) +20% fructose, 20% protein (casein), 5% fiber, 3.5% AIN-93G mineral mix, 1% vitamin mix, 0.3% L-cysteine, 0.25% choline, and 0.002% di-terc-butyl methyl phenol (HFHFr). The remaining treated groups received same composition diet except for the modulation of lipids, with 25% lard and 25% of the respective vegetable oils (olive oil, canola oil, and soybean oil), i.e., HFHFr/OL, HFHFr/CN, and HFHFr/S (Table 1).

The vegetable oils, extravirgin olive oil (®Gallo), soybean oil, and canola oil (®*Liza*) were purchased at the local market. The fatty acid profiles of lard and of respective oils are listed in Table 1, as well as peroxidability index [20]. Vitamin, mineral mix, choline, and L-cystine were purchased from Rhoster (Araçoiaba da Serra, Brazil).

Food intake and weight were determined per cage (2 animals/cage) over a period of 16 weeks, and are reported as mean food intake and weight in g/day. At the end of experiment, animals were starved for 12 hours and then anesthetized with ketamine and xylazine diluted in saline at the proportion of 1 : 1:2 ml. It was administrated dose applications of 10 *µ*l/g weight each. Blood was immediately collected by cardiac puncture, left to rest at room temperature for 30 minutes, and centrifuged at 3500 rpm at 4°C for serum separation afterward. Serum was stored at −80°C for later analysis. Liver, epididymal adipose tissue, and retroperitoneal adipose tissue were weighed and frozen in aluminum parts for further analysis.

### 2.2. Hepatic Histology

Liver fragments were fixed in 10% buffered formalin for 24 hours and embedded in paraffin. Histological preparations containing 5 *µ*m thick sections were stained with hematoxylin and eosin (H&E) for semiquantitative assessment of steatosis and/or steatohepatitis on 10 representative microscope fields at 40× magnification by a pathologist, who was blind to the samples. All divergent results were analyzed and discussed using a system of co-observer microscopic analysis [21].

### 2.3. Biochemical and Hepatic Analyses

Serum triacylglycerols (TAG), total cholesterol (TC), and cholesterol present in high-density lipoprotein (HDL) were determined using commercial kits (Labtest Diagnóstica S.A., Vista Alegre, Brazil), and very low-density lipoprotein (VLDL) cholesterol was then calculated. The same kits were used for the hepatic determinations as well. Total hepatic fat was extracted by adapted Bligh and Dyer method [22], and total TAG and total hepatic cholesterol levels were determined using commercial kits.

A direct transesterification method was applied to determine the hepatic fatty acid profile [23]. Fatty acid methylated esters were separated by gas chromatography (Shimadzu Europe, Duisburg, Germany) using an instrument equipped with an AOC-20i self-injector (Shimadzu Europe, Duisburg, Germany) and a SP-2560 fused silica column (100 m, 0.25 mm I.D, film thickness 0.20 *μ*m). Helium was used as the carrier gas. Synthetic air was used as the flame ionization detector at 250°C. Injections were performed in split mode and the time of fatty acid retention was determined by comparison with an external standard (Supelco 37 component FAME Mix).

### 2.4. Analysis of Glycemia and Insulin Resistance

Animals glycemia was determined at the end of experiment using obtained samples from animal's tail and the freestyle lite Abbot®A glucometer. The triglyceride/HDL-cholesterol ratio was calculated as a predictor of insulin resistance [24].

### 2.5. Analysis of Lipid Peroxidation and Antioxidant Parameters

Hepatic malondialdehyde MDA was determined by Gerard-Monnier et al. method [25] with some modifications, as it is thoroughly described in S1 text section.

Hepatic reduced glutathione (GSH) was determined from hepatic tissue by Sedlak and Lindsay method [26] with adaptations, and vitamin A (*α*-tocopherol) was determined by adapted Arnaud et al. method [27]. Complete methodology is detailed and described in S1 text section.

### 2.6. Statistical Analysis

One-way analysis of variance (ANOVA) was applied to the data of the various groups, followed by the Tukey post-test, using the GraphPad Prism software, version 5.00 for Windows (GraphPad Software, San Diego, CA, USA), with the level of significance set up at *p* < 0.05. Data are reported as mean ± standard deviation.

## 3. Results

### 3.1. Effects of MUFAs and PUFAs on Body Weight Gain, Food Intake, and Energy Intake

HFHFr animals' body weight started to increase significantly compared to control from 5th week on (*p* < 0.05). From 9th week, HFHFr animals' body weight increased significantly compared to control (35.00 ± 4.30 g/animal vs. 29.80 ± 2.48 g/animal, *p* < 0.01) and to treated groups (HFHFr/OL: 30.00 ± 1.89 g/animal; HFHFr/CN: 31.20 ± 2.34 g/animal; HFHFr/S: 30.63 ± 2.66 g/animal; *p* < 0.05). At the end of the experiment (16 weeks), HFHFr group had a greater body weight compared to control and treated groups (HFHFr: 44.20 ± 2.34 g/animal vs. Control: 34.80 ± 3.45 g/animal; *p* < 0.001; HFHFr/OL: 35.40 ± 4.19 g/animal; HFHFr/C: 36.10 ± 3.92 g/animal; HFHFr/S: 36.25 ± 5.70 g/animal; *p* < 0.01), suggesting that the oils were effective in maintaining body weight. There was some variation in mean food intake throughout the experimental period due to control animals ingesting more food and HFHFr animals ingesting less food. Among the treated groups, HFHFr/OL group ingested more food than any of them. Energy intake data were positively correlated with food intake data, whereas control and HFHFr groups showed lower energy intake throughout the experiment, the experimental groups, particularly HFHFr/OL group, showed greater energy intake. However, these variations were not expressed by the efficacy of diet in favoring weight gain as determined by the feed efficiency rate (FER), which only differed between control and HFHFr animals. The results are shown in Table 2.

### 3.2. Liver  Weight  and Epididymal  and Retroperitoneal  Adipose Tissue Weight and Their Relationship with Body Weight

Hepatic tissue weight was increased in the HFHFr group compared to control. Among treated groups, the PUFA-rich group (soybean oil) showed a significant reduction of hepatic tissue compared to the HFHFr group (*p* < 0.05), even though no significant reduction was observed in relation to body weight throughout the experiment. The HFHFr/CN group showed a reduction of retroperitoneal adipose tissue compared to the HFHFr group (*p* < 0.05), although there was no difference in relation to body weight. The sum of epididymal + retroperitoneal weight was higher for the HFHFr group compared to control (*p* < 0.05), but it did not differ from that of treated groups. No difference in epididymal adipose tissue or its relation to body weight was observed over 16 weeks of the experiment. The results are shown in Table 3.

### 3.3. Effects of Vegetable Oils on Triacylglycerol, Cholesterol, VLDL, HDL-C, Glycemia, and Triacylglycerol/HDL-Cholesterol Ratio

**Figure 1:**
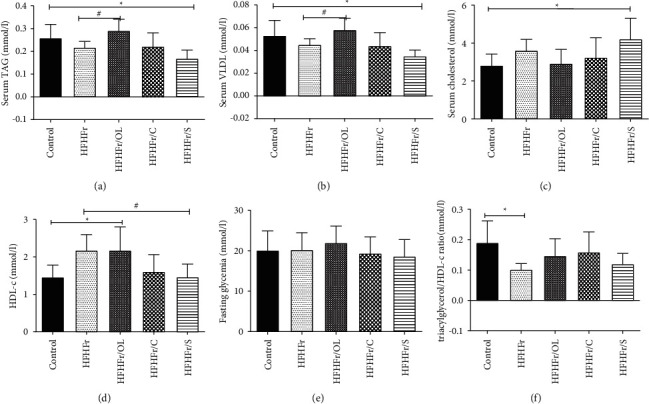
Analysis of lipid profile. Triacylglycerol (a). VLDL (b). Total cholesterol (c). HDL-C (d). Fasting glycemia (e). Triacylglycerol/HDL-C ratio (f). Data are reported as mean ± standard deviation for a period of 16 weeks. ^*∗*^*p* ≤ 0.05 vs. control; ^#^*p* ≤ 0.05 vs. HFHFr.

Serum triacylglycerol levels of the HFHFr/OL group were significantly higher than those of HFHFr group (0.291 ± 0.049 vs.0.126 ± 0.027), whereas those of HFHFr/S group were lower than control (0.168 ± 0.037 vs. 0.258 ± 0.060) (Figure 1(a)). The same results were observed for VLDL values (Figure 1(b)). HFHFr/S group showed a higher total cholesterol level than control (4.208 ± 1.102 vs. 2.818 ± 0.599) (Figure 1(c)). HDL-C levels were higher for HFHFr/OL and HFHFr groups compared to control (2,175 ± 0.624 vs. 2.175 ± 0.420 vs. 1.457 ± 0.321), while they were lower in soybean oil group compared to HFHFr group (Figure 1(d)). Glycemia and the marker of insulin resistance (triacylglycerol/HDL-C ratio) did not differ among groups (Figures 1(e) and 1(f)). About treated groups, the HFHFr/CN group was the only one that did not show changes in serum parameters.

### 3.4. The MUFAs and PUFAs of Vegetable Oils Reduced the Accumulation of Hepatic Fat

**Figure 2:**
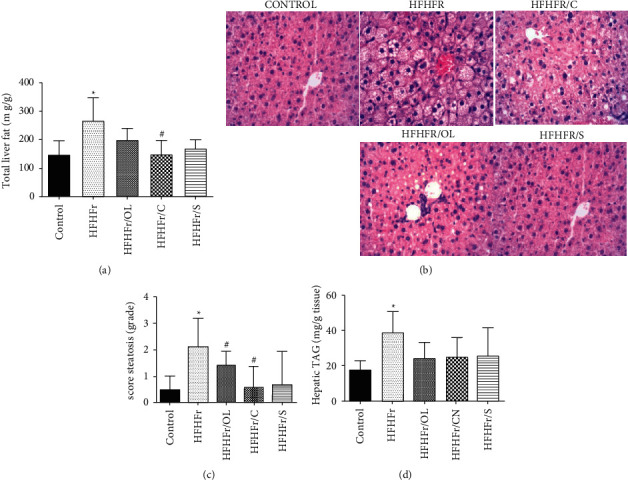
Analysis of hepatic parameters. Percent total hepatic fat (a). Liver photomicrograph (b). Steatosis score (c). Hepatic triacylglycerols (d). Data are reported as mean ± standard deviation for a period of 16 weeks. ^*∗*^*p* ≤ 0.05 vs. control; ^#^*p* ≤ 0.05 vs HFHFr.

**Figure 3:**
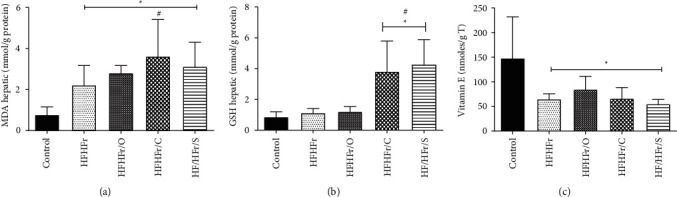
Hepatic content of MDA, GSH, and vitamin E in investigated animals (a). Hepatic GSH (b). Hepatic vitamin E (c). Data are reported as mean ± standard deviation for a period of 16 weeks. ^*∗*^*p* ≤ 0.05 vs. control; ^#^*p* ≤ 0.05 vs. HFHFr.

As expected, the western diet (HFHFr) increased the accumulation of total hepatic fat compared to control (*p* < 0.01). Among treated groups, the group receiving canola oil, mainly rich in MUFAs and PUFAs, showed a reduction in total hepatic fat accumulation compared to HFHFr group (*p* < 0.01) (Figure 2(a)). Therefore, these data were positively correlated with the hepatic histology findings. HFHFr group exhibited moderate and severe diffuse microvesicular hepatic steatosis (Figure 2(b)). Groups that received a diet rich in MUFAs and PUFAs showed reduced steatosis compared to HFHFr group. Whereas HFHFr/OL group exhibited mild to moderate macro and microvesicular steatosis, HFHFr/CN and HFHFr/S groups exhibited mild steatosis. No group showed fibrosis or inflammation (Figure 2(b)), as it can be seen in Figure 3(c) based on the NAFLD score. Hepatic triacylglycerol levels were higher in the groups receiving a western diet compared to control (38.48 ± 16.86 vs. 17.28 ± 5.4, *p* < 0.01). Although the groups receiving diets rich in MUFAs and PUFAs showed reduced triacylglycerols, the difference was nonsignificant (Figure 2(d)). Hepatic cholesterol did not show changes among groups.

### 3.5. Effects of Vegetable Oils on Oxidative Stress Parameters

Oxidative stress, final resulted product of lipid peroxidation, was assessed on the basis of the levels of MDA. Hepatic MDA was increased in all treated groups compared to control (control: 0.772 ± 0.377 vs. HFHFr: 2.212 ± 0.962 vs. HFHFr/OL: 2,805 ± 0.364 vs. HFHFr/CN: 3.613 ± 1.794 vs. HFHFr/S: 3.122 ± 1.188). In comparison with HFHFr group, only the one receiving diet rich in canola oil showed increased hepatic MDA levels (*p* < 0.05) (Figure 3(a)). Antioxidant protection was assessed on the basis of hepatic GSH levels, which were found to be higher in groups HFHFr/CN and HFHFr/S, preventing oxidative stress compared to control and to the group receiving western diet (control: 0.856 ± 0.340 vs. HFHFr: 1.121 ± 0.275; vs. HFHFr/CN: 3.808 ± 1.984 vs. HFHFr/S: 4.254 ± 1.623) (Figure 3(b)). Vitamin E levels were significantly reduced in all treated groups compared to control (*p* < 0.05) (Figure 3).

### 3.6. Effect of the Diets on the Composition of Hepatic Fatty Acids

The diet rich in omega-9 (olive oil group) and the diet rich in omega-6 (soybean oil group) increased the levels of palmitic acid (C16 : 0) compared to the group receiving western diet (HFHFr) (*p* < 0.05), with no changes in C18 : 0 levels or in the total SFA sum. C16 : 1 levels were higher in the control group than in any other group (*p* < 0.001), while there was no difference with regard to C18 : 1 or oleic acid (omega-9) levels or the total sum of fatty acids among all groups. Whereas an olive oil-rich diet reduced the hepatic levels of omega-6 (C18 : 1 n-6) compared to control (14.41 ± 1.43 vs. 21.90 ± 3.92), soybean oil-rich diet resulted in increased hepatic levels of C18 : 1 n-6 compared to control and to HFHFr group (HFHFr/S: 29.63 ± 1.44 vs. HFHFr: 18.19 ± 1.55 vs. control: 21.90 ± 3.92, *p* < 0.05). Omega-3 hepatic incorporation was reduced in both HFHFr/OL and HFHFr groups compared to control (*p* < 0.05), whereas it was increased in groups receiving canola oil (HFHFr/CN) and soybean oil (HFHFr/S) compared to control and to the HFHFr group (*p* < 0.05). Arachidonic acid levels were lower in the treated groups than in control (*p* < 0.05). Despite EPA (C20 : 5 n-3) was significantly increased in the groups receiving the canola oil and soybean oil diets compared to control (*p* < 0.05), it was only increased in the canola oil group compared to the HFHFr group (*p* < 0.001). Although DHA (C22 : 6 n-3) levels were higher in canola oil group, they did not differ significantly. The group receiving soybean oil had higher amounts of PUFAs compared to control (*p* < 0.05). The results are shown in Table 4.

## 4. Discussion

Even though it is well known that the MUFAS and PUFAS, in special long chain *n*-3 PUFAs (EPA and DHA), can affect hepatic metabolic processes, their precise effects across different physiological situations are not fully understood. Thus, not only is the quantity of ingested fats but also the fatty acids composition is of pivotal importance for human health. Here both contributed to determining the effects of lipid modulation in different commercial vegetable oils [28–32].

Saturated fat was used to modulate the set diet in this study, more specifically lard-rich in oleic acid (C18 : 1 n-9, 36, 92%), which is the featured representative of monounsaturated fatty acids, followed by SFAs, palmitic acid in particular (C16 : 0.23, 86%). As expected, extravirgin olive oil had greater final sums of MUFAs, in which the most abundant one is the oleic acid (C18 : 1 n-9, 69.82%), followed by saturated fatty acids (13%), (C18 : 3 n-3; 1.14%), as it has been demonstrated by others as well [33]. Soybean oil is rich in C18 : 2 n-6 (57.52%), MUFAs (C18 : 1 n-9, 23.25%), and omega-6 (22%), which has been also reported in literature [13]. Finally, canola oil is rich in C18 : 1 n-9 (60.01%) followed by PUFAs (19.7% C18 : 2 n-6 and 8.02% C18 : 3 n-3, especially in EPA *e* DHA) as well as MUFAS, n-9, in agreement with reported data so far [34].

Meanwhile, the three vegetable oils used in the present study yielded positive results regarding the metabolic syndrome-related parameters. The HFHFr group showed an increase in weight compared to control and the remaining groups. The animals treated with the respective oils maintained a gradual and homogeneous weight gain similar to control. Food intake varied throughout the experiment and it was positively correlated with energy intake. However, diet intake affected weight gain only in the group receiving western diet, whereas this did not occur in the groups receiving PUFAs and MUFAs, as determined by the diet efficiency rate [35].

Different types of fatty acids have different oxidation and deposition rates that may contribute to fat accumulation and weight gain. Reports have suggested positive effects of monounsaturated fatty acids (MUFAs) on weight control by increasing postprandial fat oxidation and diet-induced thermogenesis compared to SFAs or n-3 polyunsaturated fatty acids (PUFAs; flaxseed oil-rich in linolenic acid) [36]. Fatty acids regulate lipogenesis through various transcription factors and nuclear receptors, including PPARs, hepatocyte nuclear factor (HNF)-4, liver *X* receptor (LXR), and sterol response element-binding proteins (SREBPs) [37].

Moreover, a previous study showed that oil with a high P/S ratio was relevant to lower body fat accumulation, and high-MUFA oil with a high P/S ratio (HMHR; consisting of 60% MUFAs from the total fatty acids with a ratio of 5) may prevent HFD-induced increased in body weight and body fat. Therefore, quantity of ingested fats, as well as composition of fatty acids, feature a pivotal role in human health [36].

PUFAs affect essential fatty acids intake and adipose tissue lipolysis. The present findings for the groups receiving different oils demonstrate that there was no change in epididymal adipose tissue. Even though retroperitoneal adipose tissue was lower in the group receiving the canola oil diet, animals receiving western diet developed a greater volume of retroperitoneal adipose tissue and sum of adipose tissues. According to the chromatography findings in this study, canola oil is rich in n-3, especially in regard to the major incorporation of EPA and DHA. Such fatty acids regulate lipogenesis through various transcription factors and nuclear receptors, including PPARs. As it is known, PPAR is highly expressed in white adipose tissue, and its activation plays a key role in adipocyte development and differentiation resulting in lipolysis as consequence [36].

Several studies have demonstrated that omega-6 and omega-3 fatty acids suppress lipogenesis and NAFLD development by inhibiting SREBP1c and transcriptional genes involved in lipogenesis [13, 38–42]. In the present study, it was the HFHFr group that showed greater liver weight as well as greater accumulation of total hepatic fat and of hepatic triacylglycerols. It was the HFHFr/S group that otherwise featured lower accumulation of total hepatic fat and also lower steatosis score. Nevertheless, these data were positively correlated with histological analysis, as in accordance with macro and micro vesicular steatosis the present steatosis was classified as mild in HFHFr/S and HFHFr/CN groups and as moderate in HFHFr/OL group. The therapeutic effects of low dietary omega 6 : 3 ratio have been observed in animal models and in clinical trials with NAFLD/NASH patients. The significance of balanced omega 6 : 3 ratio was more evident in recent trials in which treatment of NAFLD patients with omega-3 fatty acids, such as eicosapentaenoic acid (EPA) or DHA, reduced steatosis [37].

According to Hijona et al. [43], the biochemical quantitation of hepatic fat shows a good correlation with the histological classification of hepatic steatosis proposed by Kleiner and Brunt [44]. The HFHFr/OL group showed higher serum levels of triacylglycerols and VLDL compared to the HFHFr groups, whereas the soybean oil group showed lower levels. Studies conducted on rodents have shown that diets enriched with extravirgin olive oil increase the activity of both acetyl-CoA carboxylase and fatty acid synthase and also reduce the activity of carnitine palmitoyltransferase, thus increasing lipogenesis and reducing *β* oxidation [45]. Furthermore, the reduced soybean oil values may be associated with the action of PUFAs (n-6), present in greater quantities in this vegetable oil, in terms of either both lipogenesis suppression and regulation of PPR*ɑ* expression, with the consequent degradation of fatty acids [13]. Conversely, the group receiving olive oil displayed greater quantities of HDL-C. In a study carried out with 200 volunteers, Covas et al. [45] demonstrated that the consumption of extravirgin olive oil increased the plasma levels of HDL-C. Fasting glycemia and the insulin predicting factor did not differ among the groups studied. Yan et al. [46] also found no significant difference among groups in a study that compared different proportions of *n-*6/*n-*3. In the present study, canola oil did not show significant changes in the biochemical parameters among the treated groups.

The present data demonstrated a greater quantity of palmitic acid in the HFHFr/OL and HFHFr/S groups. It may be a fact related to the aforementioned described findings, with greater steatosis in the olive oil group and an increase in serum cholesterol in the soybean oil group. Palmitic acid is known for promoting increased inflammation of hepatic cells [47]. Although the difference was not statistically significant, oleic acid levels were higher in the group receiving olive oil though. The olive oil presented lower amount of serum cholesterol. These results indicate that the molecular mechanism, through EVOO extracts, promotes a hypocholesterolemic effect through HMGCoAR activity modulation, a crucial enzyme in cholesterol biosynthesis and also the well-known target of statins [48].

Both canola oil and soybean oil induced higher EPA levels compared to control, even though these levels were higher only in the canola oil group than in the HFHFr group, while DHA showed no changes. Morrison et al. demonstrated that EPA was more effective than DHA regarding the parameters of steatosis [47]. Hanke et al. [49] demonstrated an attenuation of NAFLD through use of different quantities of canola oil and a greater hepatic quantity of EPA and DHA, alongside a reduction of inflammatory parameters. The present findings show that all the oils used for various groups' treatment increased lipid peroxidation, as demonstrated by the analysis of hepatic MDA. However, in relation to antioxidant protection based on GSH determination, an increase in hepatic GSH levels was only observed in groups treated with canola oil and soybean oil, while vitamin *E* levels were lower in the treated groups.

## 5. Conclusions

In summary, lipid modulation in a vegetable oil contained diet was able to reserve a number of adverse metabolic effects of high fat diet and high fructose diet, in special the usage of soybean oil and of canola oil. Therefore, canola oil is suggested to be useful to treat metabolic syndrome comorbidities and NAFLD. This appears to be an interesting low-cost and easily applicable alternative for the prevention/treatment of steatosis induced by rich western style diet in either or both patients with NAFLD and metabolic syndrome.

## Figures and Tables

**Table 1:** Fatty acid composition of the lard and oils used in the diets.

Fatty acids	Lard	Olive oil	Canola oil	Soybean oil
Saturated fatty acids				
C16 : 0	23.86	9.53	4.50	0.01
C18 : 0	10.89	2.85	2.29	0.36

Monounsaturated fatty acids				
C16 : 1	1.96	0.75	0.29	11.57
C18 : 1 n-9	36.92	69.82	60.01	23.15

Polyunsaturated fatty acids				
C18 : 2 n-6	20.69	6.56	19.73	57.52
C18 : 3 n-3	1.04	1.14	8.02	5.20
C20 : 4 n-3	0.07	0.09	0.05	0.01
C20 : 5 n-3	0.02	0.10	0.33	0.42
C22 : 6 n-3	0.10	0.08	0.03	0.01

Total type of fats				
∑SFA	37.16	13.08	7.91	1.10
∑MUFAs	39.59	72.72	61.92	37.07
∑PUFAs	24.29	14.18	30.15	63.81
∑n-6	20.95	10.14	20.62	57.55
∑n-3	2.15	4.03	9.49	6.22
n-6/n-3 ratio	9.74	2.52	2.17	9.25
SFA/PUFA ratio	1.53	0.92	0.26	0.02
PI of the diets	64.52	77.00	79.60	89.00

SFA, saturated fatty acids; MUFAs, monounsaturated fatty acids; PUFAs, polyunsaturated fatty acids; PI, peroxidation index. Values are reported as mean mol percent of the total fatty acid methyl esters.

**Table 2:** Final body weight, body weight gain, food intake, energy intake, and feed efficiency rate.

	Control	HFHFr	HFHFr/OL	HFHFr/CN	HFHFr/S
Final body weight (g)	34.7 ± 3.43	44.10 ± 5.66^*∗*^	35.2 ± 4.54^#^	35.8 ± 4.11^#^	36.75 ± 36.29^#^
∆ body weight gain (g)	14.40 ± 3.20	23.50 ± 5.48	14.20 ± 4.02	16.30 ± 3.59	18.00 ± 6.92
Food intake (g/week)	24.44 ± 1.36	18.29 ± 1.36^*∗*^	22.30 ± 2.14^#^	19.97 ± 0.976	20.28 ± 1.40
Energy intake (kcal/week)	96.55 ± 5.39	111.8 ± 8.34^*∗*^	136.2 ± 13.11^#^	122.0 ± 5.97	123.9 ± 8.55
Feed efficiency rate (D)	025 ± 0.007	0.045 ± 0.038	0.033 ± 0.012	0.039 ± 0.018	0.054 ± 0.017

Data are reported as mean ± standard deviation. ^*∗*^*p* ≤ 0.05 vs. control; ^#^*p* ≤ 0.05 vs. HFHFr. Groups: control; HFHFr, high fat and high fructose; HFHFr/OL, high fat and high fructose and 25% lard and 25% olive oil; HFHFr/CN, high fat and high fructose and 25% lard and 25% canola oil; HFHFr/S, high fat and high fructose and 25% lard and 25% soy oil.

**Table 3:** Phenotypic comparison of C57/BL mice fed the chow, HFHFr, HFHFr/OL, HFHFr/CN, and HFHFr/S diet for 16 weeks.

Parameters	Control	HFHFr	HFHFr/OL	HFHFr/CN	HFHFr/S
Liver weight (g)	1.25 ± 0.20	1.73 ± 0.51^*∗*^	1.35 ± 0.22	1.38 ± 0.23	1.28 ± 0.35 ^#^
Liver weight (%BW)	3.50 ± 0.38	3.8 ± 0.73	3.80 ± 0.62	3.80 ± 0.32	3.4 ± 0.44
Epididymal adipose tissue (g)	1.39 ± 0.47	2.15 ± 0.41	1.63 ± 0.71	1.57 ± 0.51	1.84 ± 0.86
Epididymal adipose tissue (%BW)	3.99 ± 1.17	4.92 ± 1.07	4.49 ± 1.75	4.29 ± 1.08	4.89 ± 1.77
Retroperitoneal adipose tissue (g)	0.35 ± 0.09	0.56 ± 0.13^*∗*^	0.41 ± 0.20	0.36 ± 0.12 ^#^	0.43 ± 0.18
Retroperitoneal (%BW)	1.00 ± 0.19	1.27 ± 0.30	1.16 ± 0.53	1.00 ± 0.27	1.15 ± 0.40
Adipose tissue sum (g)	1.75 ± 0.54	2.71 ± 0.52^*∗*^	2.04 ± 0.89	1.94 ± 0.62	2.27 ± 1.07
Adipose tissue sum (%BW)	4.99 ± 1.31	6.19 ± 1.31	5.65 ± 2.21	5.30 ± 1.32	6.05 ± 2.14

Values are reported as mean ± standard deviation (*n* = 10 per group). Control; HFHFr; HFHFr/OL; HFHFr/CN; HFHFr/S. ^*∗*^*p* ≤ 0.05 vs. control; ^#^*p* ≤ 0.05 vs. HFHFr. Groups: control; HFHFr, high fat and high fructose; HFHFr/OL, high fat and high fructose and 25% lard and 25% olive oil; HFHFr/CN, high fat and high fructose and 25% lard and 25% canola oil; HFHFr/S, high fat and high fructose and 25% lard and 25% soy oil.

**Table 4:** Liver' fatty acid composition (wt% of total methyl esters).

Fatty acids	Control	HFHFr	HFHFr/OL	HFHFr/CN	HFHFr/S
C14 : 0	0.44 ± 0.05	0.32 ± 0.07	0.23 ± 0.06^*∗*^	0.22 ± 0.07^*∗*^	0.21 ± 0.05^*∗*^
C16 : 0	27.38 ± 1.39	25.85 ± 1.35	23.09 ± 0.58^*∗*^^#^	22.43 ± 0.38^*∗*^^#^	23.72 ± 0.32^*∗*^^#^
C18 : 0	6.56 ± 1.78	5.76 ± 3.40	6.89 ± 2.76	8.67 ± 4.23	7.04 ± 2.82
∑SFA	36.36 ± 2.04	33.56 ± 3.66	31.51 ± 2.88	33.03 ± 4.35	32.99 ± 2.45

Monounsaturated fatty acids (MUFAS)					
C16 : 1	3.21 ± 0.63	1.15 ± 0.33^*∗*^	0.89 ± 0.28^*∗*^	0.66 ± 0.30^*∗*^	0.65 ± 0.15^*∗*^
C18 : 1 n-9	25.43 ± 7.97	36.17 ± 8.73	40.57 ± 7.27^*∗*^^#^	33.67 ± 11.03	23.53 ± 4.87
∑MUFAs	29.49 ± 8.61	38.28 ± 9.23	42.69 ± 8.14^*∗*^^#^	35.27 ± 11.78	24.94 ± 5.21

Polyunsaturated fatty acids (PUFAS)					
C18 : 2 n-6	21.90 ± 3.92	18.19 ± 1.55	14.31 ± 1.43^*∗*^	18.48 ± 2.64	29.64 ± 1.44^*∗*^^#^
18 : 3 n-3	0.67 ± 0.13	0.34 ± 0.05^*∗*^	0.23 ± 0.04^*∗*^	1.23 ± 0.10^*∗*^^#^	1.00 ± 0.19^*∗*^^#^
C20 : 4 n-6	0.18 ± 0.12	0.05 ± 0.03^*∗*^	0.03 ± 0.02^*∗*^	0.03 ± 0.01^*∗*^	0.04 ± 0.02^*∗*^
EPA (20 : 5 n-3)	0.16 ± 0.05	0.10 ± 0.01	0.07 ± 0.01^*∗*^	0.33 ± 0.06^*∗*^^#^	0.18 ± 0.02^*∗*^
DHA (22 : 6 n-3)	3.35 ± 1.08	2.63 ± 1.34	3.21 ± 1.15	4.04 ± 1.52	3.24 ± 1.13
∑PUFAs	34.18 ± 7.29	28.17 ± 5.93	25.80 ± 5.33	31.70 ± 7.42	42.08 ± 2.78^*∗*^

Data are mean ± SD. Data for the five groups received different vegetable oils compared by ANOVA with Tukey's test.^*∗*^Compared with control and ^#^compared with high fatty and high fructose (*p* < 0.05). SFA, saturated fatty acids; MUFA, monounsaturated fatty acids; PUFA, polyunsaturated fatty acids; HFHFr, high fatty + fructose group; HFHFr/OL, fructose and 25% olive oil and 25% lard group; HFHFr/CN, fructose and 25% canola oil and 25% lard group; HFHFr/S, fructose and 25% soybean and 25% lard group; and control, receiving 7% soybean oil.

## Data Availability

The data used to support this study are available from the corresponding author upon request.

## Additional Points

Core tip: It is a fact that there is no guideline or more targeted treatment. Due to the increase in the consumption of ultra-processed foods, rich in saturated fat and fruit, the prevalence of disease increased. Thus, studied dietary methods through dietary modulation for the prevention of nonalcoholic liver disease in a wide scenario increased prevalence of nontransmissible chronic diseases.
